# Green Synthesis of Silver Nanoparticles Using *Ligusticum mutellina* (L.) Crantz

**DOI:** 10.3390/molecules31081279

**Published:** 2026-04-14

**Authors:** Valentina Pavić, Lidija Kalinić, Zvonimir Užarević, Elvira Kovač-Andrić, Ivan Ćorić, Martina Jakovljević Kovač, Elma Džemaili, Lovro Mihajlović, Vlatka Gvozdić

**Affiliations:** 1 Department of Biology, University of Osijek, Cara Hadrijana 8A, 31000 Osijek, Croatia; vpavic@biologija.unios.hr (V.P.); lkalinic@biologija.unios.hr (L.K.); lmihajlovic@biologija.unios.hr (L.M.); 2 Faculty of Education, University of Osijek, Cara Hadrijana 10, 31000 Osijek, Croatia; zuzarevic@foozos.hr; 3 Department of Chemistry, University of Osijek, Cara Hadrijana 8A, 31000 Osijek, Croatia; eakovac@kemija.unios.hr (E.K.-A.); dzemailielm@gmail.com (E.D.); 4 Department of Medicinal Chemistry, Biochemistry and Clinical Chemistry, Faculty of Medicine in Osijek, University of Osijek, Josipa Huttlera 4, 31000 Osijek, Croatia; icoric@mefos.hr; 5 Faculty of Food Technology Osijek, University of Osijek, Franje Kuhača 18, 31000 Osijek, Croatia; mjakovljevic@ptfos.hr

**Keywords:** *Ligusticum mutellina* (L.) Crantz, silver nanoparticles, green synthesis, FT-IR, PXRD, TEM, antibacterial activity

## Abstract

Green synthesis is an eco-friendly, simple, and cost-effective process for the synthesis of metal nanoparticles from plant extracts that are rich in bioactive compounds. In the current study, the antioxidant potential and total soluble polyphenol content (TPC) of different parts of *Ligusticum mutellina* (L.) Crantz were evaluated using DPPH (2,2-diphenyl-1-picrylhydrazyl) and FRAP (ferric reducing antioxidant power) assays, and the results indicated that the seed extract was the most active plant part. HPLC analysis indicated the presence of phenolic compounds such as gallic acid, protocatechuic acid, and catechin, which may contribute to the reduction and stabilization of AgNPs. Silver nanoparticles (AgNPs) were synthesized from the aqueous seed extract of *L. mutellina*. The formation of nanoparticles was confirmed by UV–Vis spectroscopy, FT-IR analysis, powder X-ray diffraction (PXRD), and transmission electron microscopy (TEM). The UV–Vis spectrum indicated a surface plasmon resonance peak at around 411 nm, and PXRD analysis indicated an average crystallite size of around 13 nm. TEM analysis revealed predominantly spherical nanoparticles with an average size of 25.36 ± 10.76 nm. The synthesized AgNPs exhibited strong antibacterial activity against Gram-positive (*Staphylococcus aureus* and *Bacillus subtilis*) and Gram-negative (*Escherichia coli* and *Pseudomonas aeruginosa*) bacteria. Overall, the results demonstrate that *L. mutellina* seed extract represents an effective natural source of reducing and stabilizing agents for green nanoparticle synthesis and highlight the potential of the obtained AgNPs as environmentally friendly antimicrobial materials.

## 1. Introduction

Nanotechnology, the manipulation and application of materials at the nanometer scale (1–100 nm), has brought a revolution in modern science and technology, providing innovative solutions in the fields of medicine, energy, agriculture, electronics, and environmental remediation. Among the most widely investigated and commercially viable nanomaterials are silver nanoparticles (AgNPs), due to their broad-spectrum antimicrobial properties, high electrical conductivity, catalytic activity, and distinctive optical properties resulting from surface plasmon resonance (SPR) effects [1,2]. In addition to their physicochemical properties, silver nanoparticles exhibit strong antimicrobial activity through multiple mechanisms of action [3]. These include disruption of bacterial cell membranes [4], generation of reactive oxygen species (ROS) [5], interaction with thiol groups of proteins [6], and interference with DNA replication, ultimately leading to cell death [7]. Importantly, due to these multi-target mechanisms, AgNPs have shown effectiveness against a wide range of pathogenic and antibiotic-resistant microorganisms, making them promising alternatives or complements to conventional antimicrobial agents [8,9]. Recent advances in plant-mediated synthesis have further demonstrated that the biological activity of AgNPs is strongly influenced by the nature of phytochemicals present in the extract [10]. These biomolecules not only act as reducing and stabilizing agents but also affect nanoparticle size [11], morphology [12], surface chemistry [13], and ultimately their biological performance [14]. As a result, plant-derived AgNPs often exhibit enhanced antimicrobial and therapeutic properties compared to chemically synthesized counterparts [15,16]. These properties have enabled their application into antibacterial coatings, wound dressings, water purification systems, food packaging, biosensors, and drug delivery platforms. Traditional AgNPs synthesis techniques involve chemical and physical processes such as chemical reduction, electrochemical approaches, photochemical reactions, and thermal decomposition. However, these techniques involve the use of toxic reducing agents, dangerous organic solvents, and high energy input, contributing to environmental pollution and concerns about safety and sustainability [17,18]. In addition, the nanoparticles synthesized using chemical methods may have surface chemistries that are not biocompatible, thereby, limiting their direct application in biomedical fields. On the other hand, green synthesis techniques have emerged as a sustainable alternative which focuses on safety, sustainability, and resource conservation. This approach uses biological systems such as bacteria, fungi, algae, and more specifically plant extracts as reducing agents to synthesize nanoparticles in single-step, room-temperature processes that do not require the use of toxic chemicals [19,20]. Plant-mediated synthesis has received considerable attention not only due to its environmental advantages but also because it enables fine control over nanoparticle characteristics through the intrinsic biochemical composition of plant extracts [15]. Plant extracts are known to contain a wide variety of secondary metabolites such as flavonoids, phenolic acids, alkaloids, terpenoids, tannins, and proteins that have high redox potential and can reduce metal ions while simultaneously stabilizing nanoparticles and preventing aggregation [21]. Notably, the choice of plant species and its particular phytochemical composition play a significant role in determining the morphology, size distribution, and bioactivity of the resulting nanoparticles [22]. Consequently, recognizing new plant species with rich ethnobotanical and phytopharmacological traditions provides opportunities not only for the preparation functional nanomaterials but also for the rationalization and utilization of underexploited and regionally endemic plant resources. One such promising but underexploited plant species is *Ligusticum mutellina* (L.) Crantz, also known as Alpine lovage. Belonging to the family Apiaceae, *L. mutellina* is a perennial herb native to the mountainous areas of Central and Southern Europe, specifically the subalpine zones of the Alps and Dinaric Mountains. Traditionally used in Central European folk medicine, this plant has been used as a digestive stimulant and anti-inflammatory and antimicrobial agent. Previous studies have investigated its chemical composition and biological properties, and it has been found to possess a diverse phytochemical composition, including coumarins (e.g., osthole, isoimperatorin), essential oils (e.g., α-pinene, β-phellandrene, myrcene), and a variety of phenolic acids (chlorogenic, gallic, benzoic, caffeic, p-coumaric, ferulic), all of which have been recognized for their pharmacological properties [23,24,25,26,27,28,29,30,31,32].

However, there are no studies that have reported the green synthesis of metal nanoparticles from *L. mutellina* extracts. Based on the existing literature on the antioxidant activity and various bioactive compounds of this plant, the current study aims to investigate the potential of this plant as a bioresource for nanoparticle biosynthesis. The valorization of *L. mutellina* for AgNP synthesis could provide motivation for sustainable nanomaterial synthesis and help in the ethnopharmacological validation of this plant. The total soluble phenolic content and antioxidant activity of *L. mutellina* root, stem, and seeds was determined using DPPH (2,2-diphenyl-1-picrylhydrazyl) and FRAP (ferric reducing antioxidant power) assays. Furthermore, the study attempts to design a green synthesis method for AgNPs from the seed extract of *L. mutellina*. The synthesized nanoparticles were thoroughly analyzed for characterization using UV–Vis spectrophotometry, Fourier-transform infrared spectroscopy (FT-IR), powder X-ray diffraction (PXRD), and transmission electron microscopy (TEM). In addition, the antimicrobial properties of the nanoparticles were also tested against certain Gram-positive and Gram-negative bacterial strains to explore their possible applications in the biomedical field. By utilizing the phytochemical diversity of *L. mutellina*, this research effort can be considered as a contribution to the emerging area of green nanotechnology. To the best of our knowledge, this study represents the first report on the use of *Ligusticum mutellina* (L.) Crantz seed extract for the green synthesis of silver nanoparticles, thereby introducing this plant as a previously unexplored bioresource for nanoparticle production.

## 2. Results and Discussion

### 2.1. Total Soluble Polyphenol Content (TPC) and Antioxidant Activity

**Figure 1:**
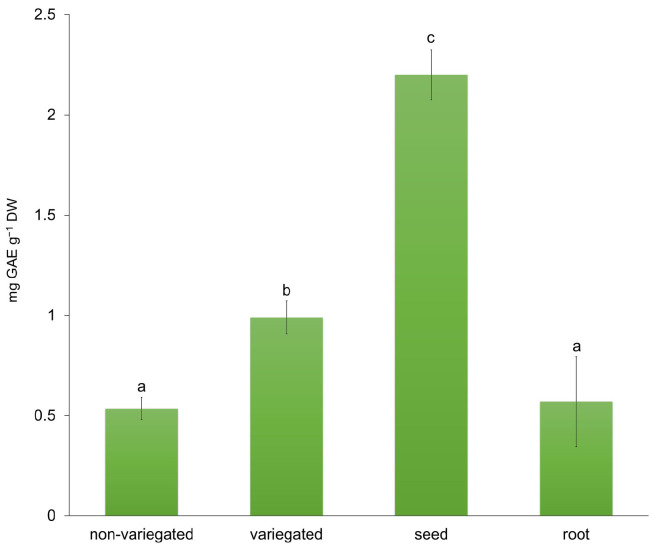
The total soluble polyphenol content (TPC) measured in the non-variegated stem, variegated stem, seeds, and root extracts of *Ligusticum mutellina*, expressed as gallic acid equivalents per g of dry weight (mg GAE g^−1^ DW). The results are presented as mean values ± standard deviation (SD). Different letters indicate statistically significant differences (*p* ≤ 0.05, one-way ANOVA followed by LSD post hoc test).

**Figure 2:**
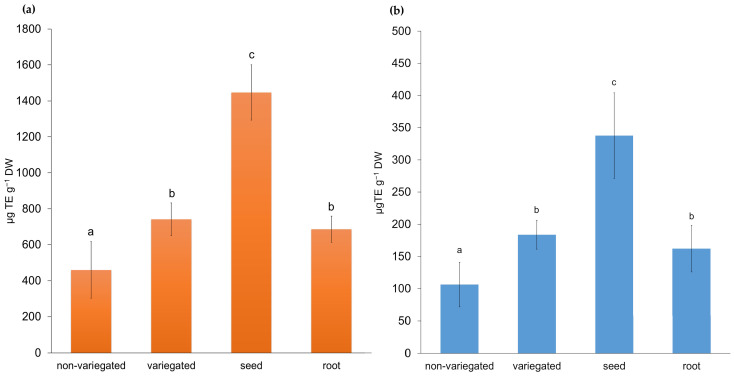
Antioxidant activity of the non-variegated stem, variegated stem, seeds and root extracts of *Ligusticum mutellina* determined by (**a**) DPPH and (**b**) FRAP assays and expressed as µg Trolox equivalents per g of dry weight (µg TE g^−1^ DW). Results are presented as mean values ± standard deviation (SD). Different letters indicate statistically significant differences (*p* ≤ 0.05, one-way ANOVA followed by LSD post hoc test).

Because the efficiency of green synthesis strongly depends on the chemical composition of plant extracts, additional characterization was performed to assess their bioactive potential. For this purpose, total soluble polyphenol content and antioxidant activity were determined. Polyphenols are well known for their strong antioxidant properties and are widely used as indicators of biological activity and medicinal value in plant materials [33]. Although not the primary focus of this research, these parameters provided valuable insights into the extract’s role in the reduction and stabilization processes involved in nanoparticle synthesis. The seeds and variegated stem had the highest total phenolic content, while the root and non-variegated stem had the lowest (Figure 1). The significant differences found between variegated and non-variegated stems can be attributed to the differences in their morphological features. Variegation is often linked to altered pigment distribution and photosynthetic efficiency, which can influence oxidative balance and, consequently, the accumulation of phenolic compounds as protective secondary metabolites. Total antioxidant activity was determined using DPPH (Figure 2a) and FRAP (Figure 2b) assays. The DPPH assay showed trends similar to those obtained by the FRAP method, although with significantly higher absolute values. The non-variegate stem exhibited significantly lower activity compared to other parts of the plant, while the seeds demonstrated the highest antioxidant activity in both assays (Figure 2).

The total soluble polyphenol content in *L. mutellina* extracts, determined using the Folin–Ciocalteu method, ranged from 0.53 ± 0.03 mg GAE g^−1^ DW (non-variegated stem) to 2.19 ± 0.05 mg GAE g^−1^ DW (seeds). These values are lower than the 15.6 mg CAE g^−1^ DW reported for methanolic extracts of the aerial parts collected during the flowering stage, as well as lower than the 6.6 mg GAE g^−1^ DW measured in aqueous seed extracts of fennel (*Foeniculum vulgare*), a related Apiaceae species [26,34]. The observed differences may be attributed to the specific plant parts analyzed and the developmental stage or timing of sampling. In particular, the samples in this study were collected in November, when the plant was no longer in the flowering stage. During this period, the synthesis of secondary metabolites, including phenolic compounds, typically declines [35]. In contrast to the study by Sieniawska et al. [26], which focused exclusively on the aerial parts of flowering plants—typically richer in bioactive compounds [36]—the present study also included underground parts (roots), as well as stems and seeds. Although the overall phenolic content was lower, the distribution of polyphenols among plant parts was consistent with patterns reported in other members of the Apiaceae family: seeds displayed the highest polyphenol concentration, followed by stems and roots [37]. A similar trend has also been reported for caraway (*Carum carvi*) and fennel (*Foeniculum vulgare*), thus proving the well-established fact that seeds contain high levels of phenolic compounds, which might be a protective mechanism during plant reproduction [20]. The findings confirm that the selected extract can be used as a good reducing and stabilizing agent. Antioxidant activity assessed by DPPH and FRAP assays demonstrated considerable activity across the analyzed plant parts, reflecting the presence of phenolic compounds and flavonoids, recognized as key antioxidants [38], in agreement with previous findings by Sieniawska et al., [30]. The highest antioxidant activity was observed in the seed extract (FRAP: 337.7 ± 66.5 µg Trolox g^−1^ DW; DPPH: 1446.7 ± 153.4 µg Trolox g^−1^ DW), which can be attributed to the higher concentration of bioactive constituents in this plant part. The elevated polyphenol content and antioxidant activity of the seed extract suggested the presence of bioactive phenolic constituents and supported its selection for nanoparticle synthesis, which was subsequently confirmed by detailed HPLC analysis.

### 2.2. HPLC Determination of Phenolic Compounds in L. mutellina Seed Extract

Since the seed extract exhibited the highest polyphenol content and antioxidant capacity, it was considered the most promising candidate for nanoparticle synthesis and was therefore subjected to further phytochemical profiling using HPLC. Although multiple reference standards were analyzed, only three phenolic acids could be reliably identified in the examined extract under the applied conditions (Table 1).

**Figure 3:**
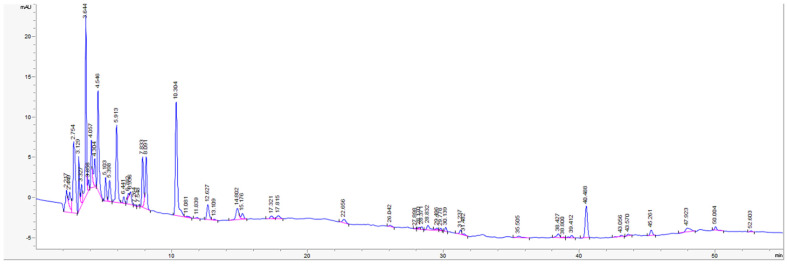
HPLC chromatogram of *L. mutellina* seed extract (270 nm).

Gallic acid, protocatechuic acid, and catechin were identified in the analyzed sample. Other phenolic acids previously reported in *Ligusticum mutellina*, such as chlorogenic, p-hydroxybenzoic, caffeic, p-coumaric, and ferulic acids [26], were not detected under the applied analytical conditions. Although several chromatographic peaks were observed in the HPLC chromatogram (Figure 3), most of these compounds could not be reliably identified, as only a limited number of compounds could be confirmed using the available reference standards under the applied analytical conditions. Therefore, the obtained results likely underestimate the overall chemical complexity of the extract, as a number of constituents remain unidentified at this stage, indicating that additional analytical approaches (e.g., LC–MS analysis) would be required for more comprehensive characterization of the extract.

### 2.3. Characterization of AgNPs

#### 2.3.1. Visual Observation and UV–Vis Spectroscopy

Previous analyses identified the highest antioxidant activity in the plant seeds; therefore, an aqueous seed extract was employed for silver nanoparticle synthesis. A stable and symmetrical SPR peak suggests effective stabilization and good colloidal stability of nanoparticles [39]. AgNPs typically exhibit an absorption maximum in the UV–Vis range between 400 and 450 nm, depending on their size, shape, composition, and surrounding medium. Because the SPR phenomenon is highly sensitive to these parameters, UV–Vis spectroscopy serves as a valuable tool for nanoparticle characterization [40,41,42,43].

**Figure 4:**
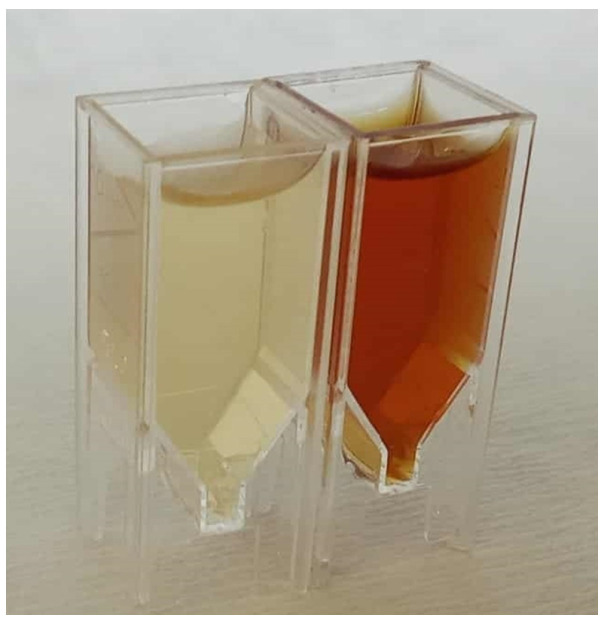
Color change in the reaction mixture before (yellow) and after the formation of AgNPs (reddish-brown).

**Figure 5:**
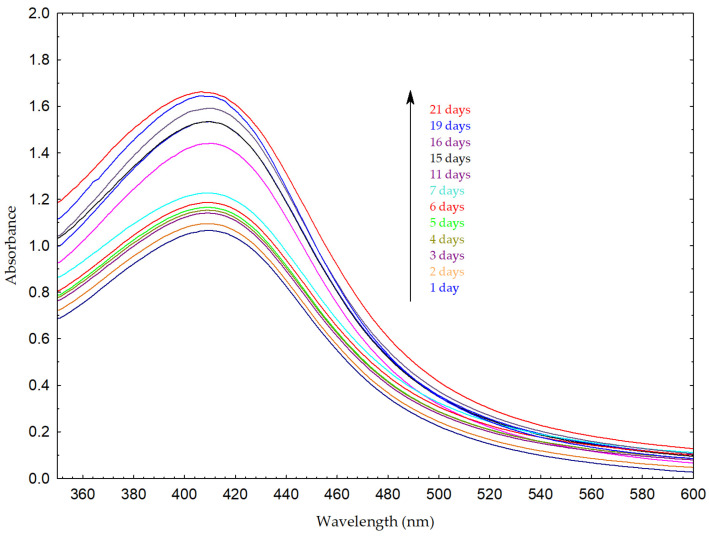
UV–Vis spectra of AgNPs synthesized using *L. mutellina* seed extract recorded over a period of 21 days. The spectra correspond to measurements taken at different time intervals, as indicated in the legend. A progressive increase in the absorbance intensity of the surface plasmon resonance (SPR) band at approximately 411 nm was observed, indicating the gradual formation of AgNPs over time. The arrow indicates the direction of increasing absorbance intensity.

The formation of AgNPs was first indicated by a visible color change in the reaction mixture from yellowish to reddish-brown (Figure 4). This observation was further confirmed by the appearance of a distinct SPR absorption maximum at approximately 411 nm, characteristic of AgNP SPR phenomenon (Figure 5) [3,44]. The formation and stability of silver nanoparticles were further monitored by UV–Vis spectroscopy over a period of 21 days. A progressive increase in the absorbance intensity of the SPR band was observed over time, suggesting the continuous reduction of silver ions and gradual generation of nanoparticles. No significant peak broadening or appearance of secondary peaks was observed, suggesting that the nanoparticle system remained relatively stable during the monitoring period. A slight shift in the SPR maximum toward shorter wavelengths was observed, which may be attributed to minor changes in particle size or the surrounding dielectric environment.

#### 2.3.2. FT-IR Analysis (Fourier-Transform Infrared Spectroscopy)

**Figure 6:**
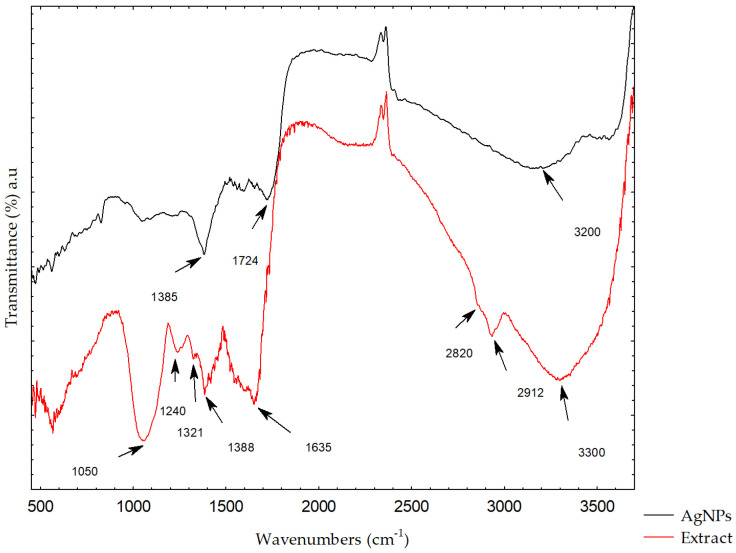
FT-IR spectra of *L. mutellina* seed extract and synthesized AgNPs.

FT-IR spectroscopy was used to determine the functional groups in the plant extract and their role in the reduction and stabilization of the silver ions in the synthesis of AgNPs [45]. Figure 6 presents the FT-IR spectra of the *L. mutellina* seed extract and the synthesized AgNPs. Changes in the spectra—such as shifts in peak positions, reductions in band intensities, disappearance of specific bands, or the emergence of new ones—indicate the participation of certain functional groups in the synthesis process. The FT-IR spectrum of the *L. mutellina* seed extract contains numerous absorption bands corresponding to bioactive constituents. The most prominent signals were observed at 3300, 2912, 2820, 1635, 1388, 1321, 1240, 1053, and 570 cm^−1^. Absorption bands characteristic of phenolic compounds, arising from O–H bending and C–O stretching vibrations, were evident within the region 1400–1220 cm^−1^, with a maximum near 1380 cm^−1^ and several weaker peaks between 1260 and 1180 cm^−1^. These features are consistent with the presence of phenolics and flavonoids, which likely contributed to the reduction of silver ions [10,45]. Comparison of the extract and AgNPs spectra revealed several characteristic changes: a slight shift and decrease in intensity of the broad band around 3300 cm^−1^ (O–H stretching of alcohols and phenolics), disappearance of the peaks at 2912 and 2820 cm^−1^ (aliphatic C–H stretching), shifts and intensity decreases in the bands at 1321, 1240, and 1053 cm^−1^ (OH in-plane bending and C–O stretching of carbohydrates), and a shift in the band near 1630 cm^−1^ attributed mainly to C=O stretching vibrations of carboxylic acid groups [46,47,48]. Additionally, the presence of a band in the low wavenumber region around 570 cm^−1^, which can be attributed to Ag–O vibrations, further supports the formation of silver nanoparticles and their interaction with oxygen-containing functional groups, as reported in previous studies [49,50]. These spectral modifications suggest that carbohydrates, flavonoids, and phenolic compounds participated in AgNPs synthesis [51,52,53]. The FT-IR results obtained in this study are consistent with previous reports on other Apiaceae species, which show similar absorption patterns, particularly the characteristic carbohydrate-associated bands in the 1000–1200 cm^−1^ region [54,55]. Additionally, the presence of a broad absorption band around 3330 cm^−1^, common in many Apiaceae extracts and associated with O–H stretching vibrations of phenolic groups, indicates the existence of functional groups that may be associated with biological activity, as reported in previous studies [56,57].

The FT-IR results are also supported by the HPLC analysis, which revealed the presence of gallic acid, protocatechuic acid, and catechin in the *L. mutellina* seed extract, all of which are well -documented for their reducing abilities [58,59,60]. These phenolic compounds have hydroxyl and aromatic groups corresponding to the absorption bands in the FT-IR spectra, particularly in the regions attributed to O–H stretching and C–O vibrations [61]. The presence of these phenolic compounds is supported by the spectral shifts observed after nanoparticle formation. These compounds are known for their strong redox properties and are likely to contribute to the reduction and stabilization of AgNPs, as supported by previous studies on plant-mediated nanoparticle synthesis. However, their specific role in nanoparticle formation in this study is inferred and not directly experimentally confirmed. Future studies involving isolated compounds or mechanistic investigations would be necessary to confirm their specific roles in nanoparticle formation. The FT-IR and HPLC analyses collectively suggest that the phenolic compounds present in the extract may contribute to nanoparticle formation and stabilization. However, their specific role in this process is inferred and not directly experimentally confirmed. The results of this study demonstrate that *Ligusticum mutellina* seed extract can serve as an effective reducing and stabilizing agent, confirming its potential as a novel and sustainable resource for the green synthesis of silver nanoparticles.

#### 2.3.3. Powder X-Ray Diffraction

**Figure 7:**
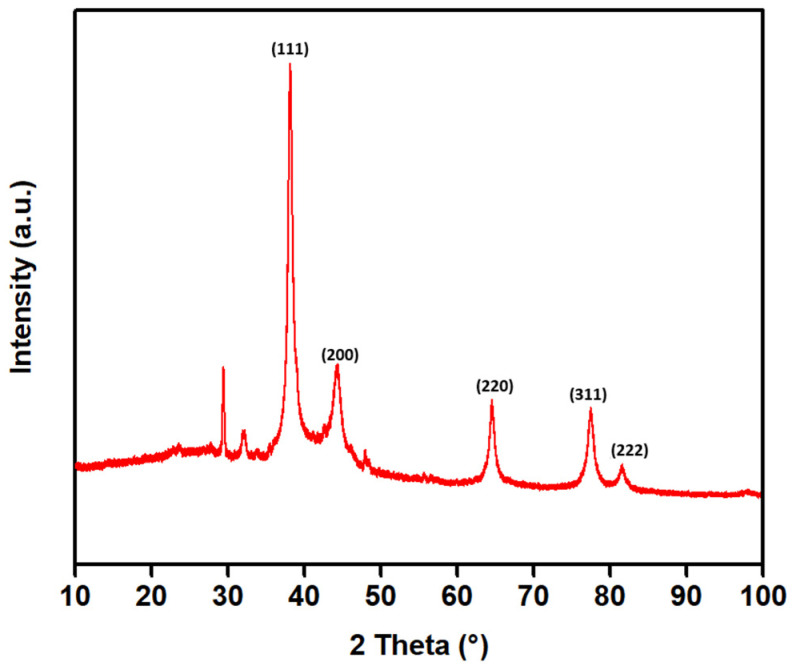
Powder X-ray diffraction (PXRD) pattern of synthesized AgNPs. The dominant diffraction peaks correspond to the (111), (200), (220), (311), and (222) planes of face-centered cubic metallic silver. The corresponding Miller indices (hkl) are indicated above the diffraction peaks.

Powder X-ray diffraction (PXRD) was used to identify the crystalline nature of the synthesized AgNPs. As green synthesis is a process involving complex mixtures of bioactive compounds that act as reducing as well as stabilizing agents, it is necessary to confirm the presence of a distinct crystalline structure. PXRD analysis yields information regarding crystallinity, crystallite size, and presence of impurities, which cannot be derived from spectroscopic analysis. Through comparison of the resulting diffraction pattern with standard reference data, PXRD facilitates the confirmation of the crystal structure and determination of the phase purity. Moreover, the width of the diffraction peaks provides information on the average crystallite size using the Scherrer equation, which is an important factor in nanoscale properties. Thus, PXRD is an important technique for confirming the formation and structural integrity of nanoparticles after synthesis [62]. The PXRD analysis revealed the crystalline structure of the synthesized silver nanoparticles (Figure 7). The diffraction pattern reveals sharp Bragg peaks at 2θ values of 38.11°, 44.16°, 64.48°, 77.40°, and 81.49°, which correspond to the (111), (200), (220), (311), and (222) planes of a face-centered cubic (fcc) lattice (space group Fm-3m) of metallic silver. The average crystallite size was calculated using the Scherrer equation:

D = (K ∙ λ)/(β ∙ cos θ),

where D is the crystallite size (nm), K is the shape factor, λ is the X-ray wavelength, β is the full width at half maximum (FWHM) of the diffraction peak, and θ is the diffraction angle. From the PXRD results and using the Scherrer equation, the average crystallite size of the synthesized AgNPs was found to be about 13 nm. The sharp and strong Bragg peaks, especially the prominent (111) peak, also support the high crystallinity and successful preparation of metallic silver nanoparticles using the *L. mutellina* seed extract. In addition to the main Ag reflections, weak and broadened features were observed in the lower angle region (≈31–33°), which may be attributed to minor contributions from silver oxide phases (Ag_2_O or AgO). These features do not correspond well to characteristic AgCl reflections, as the typical AgCl peak at ~27.5° was not observed in the obtained sample. Furthermore, the feature observed near ~29° is slightly shifted from standard AgCl positions and is more likely associated with residual organic or amorphous components originating from the plant-mediated synthesis process. As this is the first report on the synthesis of AgNPs using *L. mutellina*, the crystallite size of synthesized AgNPs was compared with those from other Apiaceae species. The size of the nanoparticles prepared in the present study (≈13 nm) is smaller than those of *Petroselinum crispum* (30–32 nm) and *Anethum graveolens* (30–35 nm) [63,64]. The smaller crystallite size observed in this study may be explained, based on literature reports, by differences in phytochemical composition and synthesis conditions [65]. Generally, smaller nanoparticles have a higher surface-area-to-volume ratio and enhanced reactivity, which may be beneficial in catalytic or biomedical applications.

#### 2.3.4. Transmission Electron Microscopy

**Figure 8:**
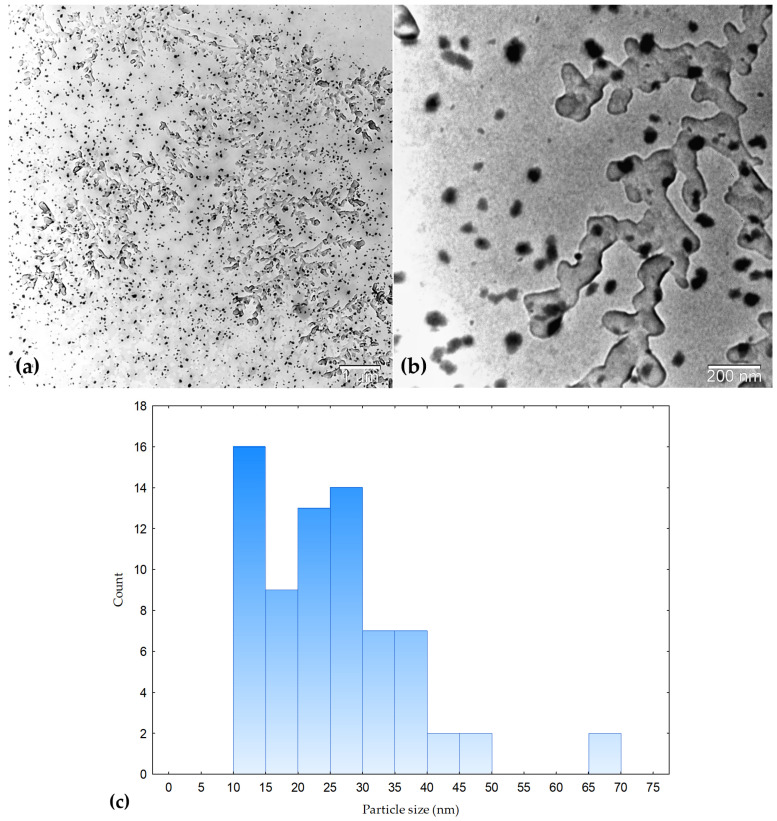
TEM micrographs of silver nanoparticles synthesized using *L. mutellina* seed extract at different magnifications: (**a**) low-magnification overview (scale bar: 1 µm) and (**b**) higher-magnification image showing individual nanoparticles and aggregates (scale bar: 200 nm). (**c**) Particle size distribution histogram obtained from measurements of clearly distinguishable individual nanoparticles using ImageJ software. The nanoparticles exhibit predominantly spherical morphology with noticeable size variation.

Transmission electron microscopy (TEM) was used to examine the morphology of the synthesized silver nanoparticles. TEM provides direct visualization of nanoparticle shape and complements other characterization techniques such as PXRD and UV–Vis spectroscopy [66]. As shown in Figure 8, the nanoparticles exhibit predominantly spherical morphology with noticeable size variation. Particle size distribution was determined by measuring clearly distinguishable individual nanoparticles using ImageJ software (version 1.54r, 25 September 2025). The average particle size was 25.36 ± 10.76 nm, with a size range of 10.32–65.27 nm. Some larger structures observed in the micrographs are attributed to agglomerated structures. The TEM results are therefore considered approximate and primarily provide qualitative insight into nanoparticle morphology. The observed particle size is larger than the crystallite size obtained from PXRD (~13 nm), which is expected since PXRD reflects crystallite size, whereas TEM reflects overall particle dimensions. Nanoparticles within this size range are generally associated with enhanced antibacterial activity due to their high surface-area-to-volume ratio and improved interaction with bacterial cells [67].

### 2.4. Antibacterial Activity of AgNPs

AgNPs synthesized using the aqueous seed extract of *L. mutellina* demonstrated pronounced antibacterial activity against both Gram-positive (*Bacillus subtilis*, *Staphylococcus aureus*) and Gram-negative (*Escherichia coli*, *Pseudomonas aeruginosa*) bacteria (Table 2). The strongest inhibitory effect was observed against *P. aeruginosa*, with a minimum inhibitory concentration (MIC) of 3.90 µg mL^−1^. This result is especially interesting, given the known natural and acquired resistance of *P. aeruginosa* to a wide variety of antibiotics, including aminoglycosides, quinolones, and β-lactam [68]. The increased susceptibility of this particular strain to the synthesized AgNPs can be ascribed to the distinctive mechanism of action of AgNPs, which include cell membrane damage, the production of reactive oxygen species, and interference with cellular metabolism, which differ from those of conventional antibiotics. The AgNPs also showed potent inhibitory activity against *B. subtilis* and *S. aureus*, both showing same MIC values of 7.81 µg mL^−1^, indicating their high potency against Gram-positive bacteria. The potency against *E. coli* was slightly lower, with an MIC of 15.63 µg mL^−1^, but still in the potent antibacterial concentration range. Taken together, these data confirm the broad-spectrum antimicrobial efficacy of the biosynthesized AgNPs.

The observed antibacterial properties evidenced in this study can primarily be attributed to the synthesized AgNPs. The phytochemical composition of the *L. mutellina* seed extract, including phenolic acids and flavonoids identified by HPLC (gallic acid, protocatechuic acid, and catechin), is likely involved in nanoparticle formation by acting as reducing and stabilizing agents. These compounds are known for their redox properties and may influence the formation of reactive nanoparticles with functionalized surfaces. Phenolic compounds have been reported to interact with bacterial membranes, affecting permeability and cellular processes, and may contribute to antibacterial effects when associated with AgNP surfaces. However, their direct contribution or potential synergistic interaction with AgNPs was not evaluated in this study and therefore cannot be conclusively determined. Future studies should include comparative evaluation of the plant extract alone and in combination with AgNPs to assess potential synergistic effects [69,70,71,72,73]. When compared with previously reported studies, the antibacterial efficacy of *L. mutellina*–mediated AgNPs is comparable to, or even higher than, that of other green-synthesized nanoparticles. For instance, Biswas et al., (2018) [74] reported MIC values ranging from 2 to 40 µg mL^−1^ for *Solanum viarum* extract-based AgNPs, while Veisi et al., (2016) [75] observed MICs of 20–25 µg mL^−1^ for nanoparticles synthesized using oak fruit bark extract. Likewise, the MIC values of 5.52–31.96 µg mL^−1^ were reported by Devanesan and Al Salhi (2021) [76] for AgNPs prepared from *Abelmoschus esculentus* flower extracts. However, the relatively lower MICs values of 3.90–15.63 µg mL^−1^ in the current study suggest higher antimicrobial activity of AgNPs prepared from *L. mutellina* seeds, particularly against *Pseudomonas aeruginosa*. The present study expands the range of plant species used in green synthesis by introducing *Ligusticum mutellina* as a novel bioresource. Compared to previously reported plant-mediated syntheses of AgNPs, the synthesized nanoparticles exhibited comparable or enhanced antibacterial activity, highlighting the potential of this plant for sustainable nanomaterial production. In contrast to conventional antibiotics, the antibacterial properties of AgNPs are realized through multiple mechanisms, such as cell membrane disruption [77], generation of oxidative stress [78,79], and interference with cellular processes [80,81]. In addition, the small particle size and large surface area of the synthesized AgNPs promote close interaction with microbial cell membranes, thereby enhancing their bactericidal efficiency, even against resistant strains [3,82,83]. The green synthesis method used in this study is a sustainable and environmentally friendly process, as it does not require the use of toxic chemicals and is carried out under mild conditions. Overall, the *L. mutellina*–mediated AgNPs exhibit strong antibacterial properties, particularly against *P. aeruginosa*, a clinically significant and highly resistant pathogen. These findings highlight their potential as sustainable and effective candidates for the development of novel antimicrobial agents. The combined results of HPLC, FT-IR, and antibacterial analyses suggest that phenolic constituents present in the *L. mutellina* seed extract may contribute to nanoparticle formation and stabilization. However, their direct role in antibacterial activity or potential synergistic effects with AgNPs were not evaluated in this study and cannot be conclusively determined. Although the present study provides comprehensive physicochemical characterization of the synthesized AgNPs, additional analyses such as zeta potential measurements would further elucidate their surface charge and long-term colloidal stability. Future studies should also focus on application-oriented investigations, including cytotoxicity, biocompatibility, and potential use in biomedical or antimicrobial formulations. The strong antibacterial activity observed in this study suggests that the synthesized AgNPs could be promising candidates for applications in antimicrobial coatings, wound dressings, or water treatment systems.

## 3. Materials and Methods

### 3.1. Plant Material

In the procedure for determining antioxidant activity, the root, stems (one exhibiting a uniform colouration and the other displaying variegation) and seeds were analyzed separately. The plant material was collected directly by the authors from the area of Poljička Kosa (Gorski kotar, Croatia; 45.3487° N, 14.8571° E) in late autumn, November 2024. The collected material was air-dried at room temperature and stored in a dry, dark place in airtight containers until further use. Prior to analysis, the plant material was processed in its dried form. The plant was identified and authenticated by Prof. Valentina Pavić and a voucher specimen (No. LM-2024-01) was deposited at the Department of Biology, University of Osijek, Osijek, Croatia.

### 3.2. Determination of Total Antioxidant Activity and Soluble Polyphenols Content

For the analysis 0.5 g of plant material was extracted with 5 mL of distilled water and kept for 24 h at room temperature with occasional agitation to allow extraction. The samples were then centrifuged for 10 min at 3000 rcf and 4 °C (Centric 322 A, Tehtnica, Železniki, Slovenia), and the obtained supernatants were used for the determination of total soluble polyphenol content (TPC) [84] and total antioxidant activity using DPPH and FRAP assays as previously described [85,86] on a microtiter plate reader (Tecan, Spark, Männedorf, Switzerland), with minor modifications. For the DPPH assay, the absorbance was measured at 517 nm after incubation for 30 min in the dark at room temperature. For the FRAP assay, the absorbance was measured at 593 nm after incubation at 37 °C for 15 min in the dark. All measurements were performed on three independent samples, each analyzed in four technical replicates (*n* = 12), and the results are expressed as mean ± standard deviation. TPC was expressed as gallic acid equivalents (GAE) per gram of dry weight, whilst for DPPH and FRAP assays, as µg of Trolox equivalents per gram of dry weight.

### 3.3. Determination of Individual Phenolic Compounds by HPLC

HPLC analysis of the *L. mutellina* seed extract was performed using an Agilent 1260 Infinity II HPLC system (Agilent Technologies, Santa Clara, CA, USA) equipped with an Intersil^®^ ODS-3 column (4.6 mm × 250 mm, 5 µm; GL Sciences Inc., Tokyo, Japan). Chromatographic separation was achieved by gradient elution at a flow rate of 1 mL min^−1^, over 55 min. The mobile phase consisted of 0.1% formic acid in milli-Q water as phase A and 0.1% formic acid in methanol (phase B). The gradient was as follows: 0–8 min: 90–75% A; 8–16 min: 75% A; 16–25 min: 75–55% A; 25–28 min: 55% A; 28–45 min: 55–20% A; 45–55 min: 20% A followed by a 20 min re-equilibration period to initial conditions. The injection volume was 10 μL. UV detection was performed at 240, 250, 260, 270, 280, 330 and 360 nm. The analysis was carried out at 50 °C. Component identification was carried out based on retention times and comparison of the UV absorption spectra in the extracts with the spectrum of the standards. Quantification was made based on external calibration. Standard stock solutions were prepared in methanol, from which seven concentrations were prepared (concentration range 10–500 mg L^−1^). The retention times for gallic acid were 6.524 min, for protocatechuic acid 10.290 min and for catechin 12.952 min. The linearity of the calibration curve was confirmed with R^2^ = 0.99904 for gallic acid, R^2^ = 1.00000 for protocatechuic acid and R^2^ = 0.99997 for catechin. Gallic acid, protocatechuic acid, and catechin standards were purchased from Sigma-Aldrich (St. Louis, MO, USA).

### 3.4. Preparation of L. mutellina Seed Extract for AgNP Synthesis

To 1.0 g of *Ligusticum mutellina* seeds, 40 mL of water was added. The beaker was covered, and the contents were stirred for 60 min at room temperature (T = 20 °C). The covered beaker was then left to stand for 48 h at room temperature (T = 20 °C), after which the extract was filtered using Whatman No. 1 filter paper. The experiment was performed in triplicate.

### 3.5. Synthesis of Silver Nanoparticles

Under slow stirring 60 mL of 3 mM AgNO_3_ solution was gradually added to 30 mL of the filtrate (2:1). The color change in the solution was monitored using UV–Vis spectrophotometer over a period of three weeks. Afterward, the mixture was centrifuged for 25 min at 6000 rpm (Centric 322 A, Tehtnica, Železniki, Slovenia). The resulting pellet was air-dried at room temperature (approximately 22 °C) for five days in the dark. The experiment was performed in triplicate.

### 3.6. UV–Vis Spectroscopy

UV–Vis spectroscopy (UV-1900, Shimadzu Corporation, Kyoto, Japan) was used to monitor nanoparticle formation. The absorption spectra were recorded in the wavelength range of 350–600 nm at regular time intervals over a 21-day monitoring period. The appearance and position of the characteristic surface plasmon resonance (SPR) band were used as indicators of AgNP formation and stability.

### 3.7. FT-IR (Fourier-Transform Infrared Spectroscopy)

FT-IR analysis was carried out using an FTIR-8400S spectrophotometer (Shimadzu Corporation, Kyoto, Japan). FT-IR spectroscopy was used to analyze the spectra of the *L. mutellina* seed extract and the synthesized AgNPs in order to identify possible spectral changes, including peak shifts and variations in band intensities.

### 3.8. Powder X-Ray Diffraction (PXRD)

PXRD analysis was performed using a Panalytical Aeris X-ray diffractometer (Malvern Panalytical Ltd., Malvern, UK) to identify the crystalline nature of the synthesized AgNPs. The diffraction peak positions and intensities were analyzed to determine the crystalline nature of the synthesized nanoparticles.

### 3.9. Transmission Electron Microscopy (TEM)

The morphology and size distribution of the synthesized AgNPs were investigated using a transmission electron microscope JEOL JEM-1200EX II (JEOL Ltd., Tokyo, Japan). The average particle diameters were determined from TEM micrographs using ImageJ software (version 1.54r, 25 September 2025). TEM analysis was used to determine the shape, size distribution, and extent of aggregation of the nanoparticles.

### 3.10. Evaluation of the Antibacterial Efficacy and Determination of MICs of Synthesized AgNPs

The antibacterial activity of silver nanoparticles synthesized using the aqueous seed extract of *Ligusticum mutellina* seeds was evaluated using a broth microdilution method, as previously described [87]. The inhibitory effects of AgNPs were tested against four standard bacterial strains obtained as MicroSwab kits (Mecconti labs Sp. z o.o., Warsaw, Poland): Gram-positive bacteria (*Bacillus subtilis* subsp. *spizizenii* WDCM 00003–ATCC^®^ 6633™ and *Staphylococcus aureus* WDCM 00032, WDCM 00193–ATCC^®^ 6538™) and Gram-negative bacteria (*Escherichia coli* ATCC^®^ 35218™ and *Pseudomonas aeruginosa* WDCM 00025–ATCC^®^ 27853™). Bacterial suspensions were prepared in Mueller–Hinton broth (Cultimed, Barcelona, Spain) and adjusted to approximately 5 × 10^5^ CFU mL^−1^, corresponding to the mid-exponential growth phase. Twofold serial dilutions of AgNPs (ranging from 1000 to 0.244 µg mL^−1^) were prepared in sterile, flat-bottom 96-well polypropylene microtiter plates (Kartell S.p.A., Noviglio (MI), Italy). Ciprofloxacin (Hospira, Hurley, Maidenhead, Berkshire, England, UK) was used as a reference antibiotic and tested under the same dilution scheme. Control wells included bacterial growth controls, solvent controls, and sterility controls to ensure experimental validity. The microtiter plates were incubated at 37 °C for 24 h. Following incubation, triphenyltetrazolium chloride (TTC, VWR Chemicals, Leuven, Belgium) was added to each well, and the plates were incubated for an additional 3 h under the same conditions. The lowest concentration of AgNPs that totally prevented apparent bacterial growth, as seen by the lack of color development, was determined as the minimum inhibitory concentration (MIC). All experiments were carried out in triplicate to ensure reproducibility of results. The MIC values were expressed in micrograms per milliliter (µg mL^−1^). Due to the consistent inhibition of growth at equal concentrations in all replicates, mean MIC values were not calculated.

## 4. Conclusions

This study describes, for the first time, the successful green synthesis of silver nanoparticles using *Ligusticum mutellina* seed extract, a plant traditionally used in folk medicine. The synthesis was achieved under mild and environmentally friendly conditions, without the use of toxic reagents, high temperatures, or additional stabilizing agents, highlighting the simplicity and sustainability of the proposed approach. Comprehensive physicochemical characterization studies, including UV–Vis spectroscopy, FT-IR, PXRD, TEM, and HPLC analysis, confirmed the formation of crystalline AgNPs. The presence of a characteristic surface plasmon resonance (SPR) band at approximately 411 nm, along with distinct diffraction peaks corresponding to a face-centered cubic silver structure, verified nanoparticle formation. TEM analysis revealed predominantly spherical nanoparticles with an average size of 25.36 ± 10.76 nm, while PXRD analysis indicated a crystallite size of approximately 13 nm. HPLC analysis indicated the presence of phenolic compounds, such as gallic acid, protocatechuic acid, and catechin, which are known for their redox properties and likely contribute to the reduction and stabilization of AgNPs. However, their specific role in nanoparticle formation remains to be further elucidated. The synthesized nanoparticles exhibited pronounced broad-spectrum antibacterial activity against both Gram-positive (*Bacillus subtilis* and *Staphylococcus aureus*) and Gram-negative (*Escherichia coli*) bacteria, with particularly strong activity against *Pseudomonas aeruginosa*, a clinically relevant and resistant pathogen. Overall, these findings highlight that *L. mutellina* seed extract represents as a promising natural source of reducing and stabilizing agents for sustainable nanoparticle production and support the potential application of plant-mediated AgNPs as environmentally friendly antimicrobial materials. Future research should include detailed evaluation of nanoparticle surface charge (e.g., zeta potential), as well as in vitro and in vivo biological effects, including cytotoxicity studies to assess their safety, biocompatibility, and potential applications in biomedical and antimicrobial systems.

## Figures and Tables

**Table 1:** Content of individual phenolic compounds in *L. mutellina* seed extract determined by HPLC.

	Concentration (µg g^−1^)		
Sample	Gallic Acid	Protocatechuic Acid	Catechin
*L. mutellina* seed extract	326.72	201.13	866.30

**Table 2:** Minimum inhibitory concentrations (MICs) of *L. mutellina*-mediated silver nanoparticles (AgNPs) against selected bacterial strains.

	MIC (µg mL^−1^)			
	*B. subtilis*	*S. aureus*	*E. coli*	*P. aeruginosa*
AgNPs	7.810	7.810	15.630	3.900
Ciprofloxacin	0.244	1.953	1.953	0.488

## Data Availability

The data presented in this study are available on request from the corresponding author.
